# EGS-Net: a knowledge-augmented machine learning framework for predicting future high-myopia risk from longitudinal school-screening trajectories

**DOI:** 10.3389/fmed.2026.1843421

**Published:** 2026-06-25

**Authors:** Zhan Tang, Na Zhao, Zhaoyu Huang, Jinhao Lu, Chao Dai, Jian Wang, Runze Zheng

**Affiliations:** 1 School of Physics, Zhejiang University, Hangzhou, China; 2 School of Software, Yunnan University, Kunming, Yunnan, China; 3 College of Information Engineering and Automation, Kunming University of Science and Technology, Kunming, Yunnan, China

**Keywords:** childhood myopia, explainable machine learning, future high-myopia risk prediction, knowledge-augmented AI, longitudinal school screening, risk stratification

## Abstract

The rapid increase in childhood myopia highlights the need for accurate, non-invasive tools for early risk stratification in large-scale screening; however, static cross-sectional data often fail to capture dynamic refractive trajectories. In this study, we developed and validated an Expert-Guided Stacking (EGS) predictive framework using longitudinal school screening data (Autumn 2023–Autumn 2025) from Binchuan County, China. For each student, predictors were constructed only from screening records preceding the outcome-defining follow-up record, thereby preserving the original temporal prediction boundary. We first evaluated performance across six conventional classifiers (LR, RF, XGBoost, SVM, NB, and AdaBoost), then proposed a hybrid EGS model that integrates a multi-model ensemble architecture with a clinical risk-heuristic override module. This framework was specifically designed to reduce false negatives and improve prediction of future high-myopia risk by leveraging historical longitudinal refractive trajectories. Model development used student-level partitioning, with 5-fold cross-validation for tuning and held-out test-set evaluation for final performance assessment. Although AdaBoost attained a high overall discrimination (AUC = 0.9992), the proposed EGS framework achieved clinically favorable utility with high Recall (0.9533) and Precision (0.9211), enabling reliable future-risk identification while avoiding the false-positive burden of less precise high-recall models. SHAP analysis verified the critical contribution of longitudinal trajectory features to model interpretability and transparency. Our findings demonstrate that this knowledge-augmented ML approach delivers a robust, scalable solution for school-based myopia surveillance, with a priority on high-risk recall to support timely clinical intervention and personalized vision care.

## 1 Introduction

Myopia has emerged as a critical global public health challenge in the 21st century. Epidemiological projections suggest that by 2050, approximately 50% of the global population will be affected by myopia, with a substantial rise in the prevalence of high myopia (1). This trend is particularly pronounced in East Asian regions, where myopia prevalence among school-leavers has reached alarming levels (2, 3). The public health implications extend far beyond the requirement for optical correction; higher degrees of refractive error are intrinsically linked to an increased risk of irreversible, sight-threatening complications, including myopic maculopathy, retinal detachment, and glaucoma (4).

Myopia is widely recognized as a multifactorial condition shaped by the complex interplay between genetic susceptibility and rapidly shifting environmental exposures (2). Contemporary lifestyle changes, characterized by intensified educational demands and prolonged near-work activities, have been consistently associated with elevated myopia risk in children (5). Conversely, evidence suggests that increased outdoor time serves as a protective factor against myopia onset and progression (6). Recent large-scale screenings during the COVID-19 pandemic further underscore the sensitivity of refractive development to lifestyle disruptions, reporting a measurable myopic shift in younger children following periods of home confinement (7).

Early detection and timely intervention are paramount for mitigating progression and preventing the transition to high myopia. School-based vision screening programs offer a scalable approach for the mass identification of at-risk children. However, the clinical “gold standard”—cycloplegic refraction—is often impractical for routine campus-wide deployment due to its invasive nature, resource requirements, and low community acceptance (8). Consequently, screening programs frequently rely on non-cycloplegic autorefraction paired with uncorrected distance visual acuity (UCDVA). Nevertheless, non-cycloplegic measures in children can be confounded by active accommodation, leading to potential misclassification, particularly in younger developmental stages where refractive status is most volatile (9).

With the advancement of artificial intelligence, machine learning (ML) models provide a robust framework to integrate multi-dimensional predictors and capture non-linear relationships (10–17). While current ML research focuses on maximizing overall accuracy, the clinical consequence of a false negative risk prediction—that is, failing to flag a child who later meets the high-myopia endpoint—is significantly more detrimental than that of a false positive. Traditional data-driven models often struggle to achieve high *Recall* for children at future high-myopia risk whose historical trajectories deviate from population averages. Furthermore, while explainable AI methods like SHapley Additive exPlanations (SHAP) improve transparency (18–21), interpretability alone does not inherently guarantee the capture of atypical, high-risk longitudinal trajectories.

Recent perspective work also emphasizes that next-generation myopia prediction should explicitly bridge genetic susceptibility, environmental exposure, and modifiable behavior, rather than treating these domains in isolation (22). Complementing this view, recent reviews in ophthalmology AI have highlighted the translational gap between algorithmic performance and real-world deployment, particularly regarding data shift, external validity, and clinician trust (23).

Despite these methodological advances, two critical gaps remain: (1) most existing models rely on cross-sectional snapshots, failing to leverage the individualized kinetic signals provided by longitudinal screening and (2) pure data-driven models lack the integration of clinical domain knowledge, which acts as a necessary “safety net” for high-risk individuals. There is an urgent need for a framework that synergizes the predictive power of ensemble learning with explicit clinical heuristics.

In this study, we propose **EGS-Net**, a knowledge-augmented ML framework developed using a longitudinal school screening dataset from Binchuan County, Yunnan Province, China. By utilizing trajectory-derived features, such as the annualized spherical equivalent (SE) progression rate, and implementing an Expert-Guided Stacking (EGS) architecture, we aim to significantly enhance predictive sensitivity for future high-myopia risk. By integrating multi-model ensemble learning with a deterministic clinical-heuristic override mechanism, this work provides a high-recall, scalable tool for personalized myopia surveillance, ensuring that children predicted to be at elevated future high-myopia risk receive timely intervention.

The overall design and information flow of the proposed framework are shown in the architecture schematic, while implementation details are provided in the Materials and Methods section.

The main contributions of this study are summarized as follows:

- **Novel hybrid architecture (EGS-Net):** We propose a knowledge-augmented framework that synergizes a multi-model Stacking ensemble with a clinical-heuristic decision engine, bridging the gap between black-box data prediction and expert clinical judgment.
- **Expert-guided override mechanism:** We implement an explicit clinical override logic based on annualized progression kinetics. This mechanism acts as a “safety net” to targetedly reduce missed future-risk identification in children at future high-myopia risk with atypical historical refractive trajectories that are often missed by standard algorithms.
- **Superior predictive sensitivity:** Through rigorous validation on a longitudinal cohort, we demonstrate that EGS-Net achieves a superior mean Recall of 0.9533. Our results show that this hybrid approach significantly outperforms standalone ML models, offering a more reliable solution for public health programs where identifying students at future high-myopia risk is the primary concern.

## 2 Materials and methods

### 2.1 Study design and population

**Figure 1:**
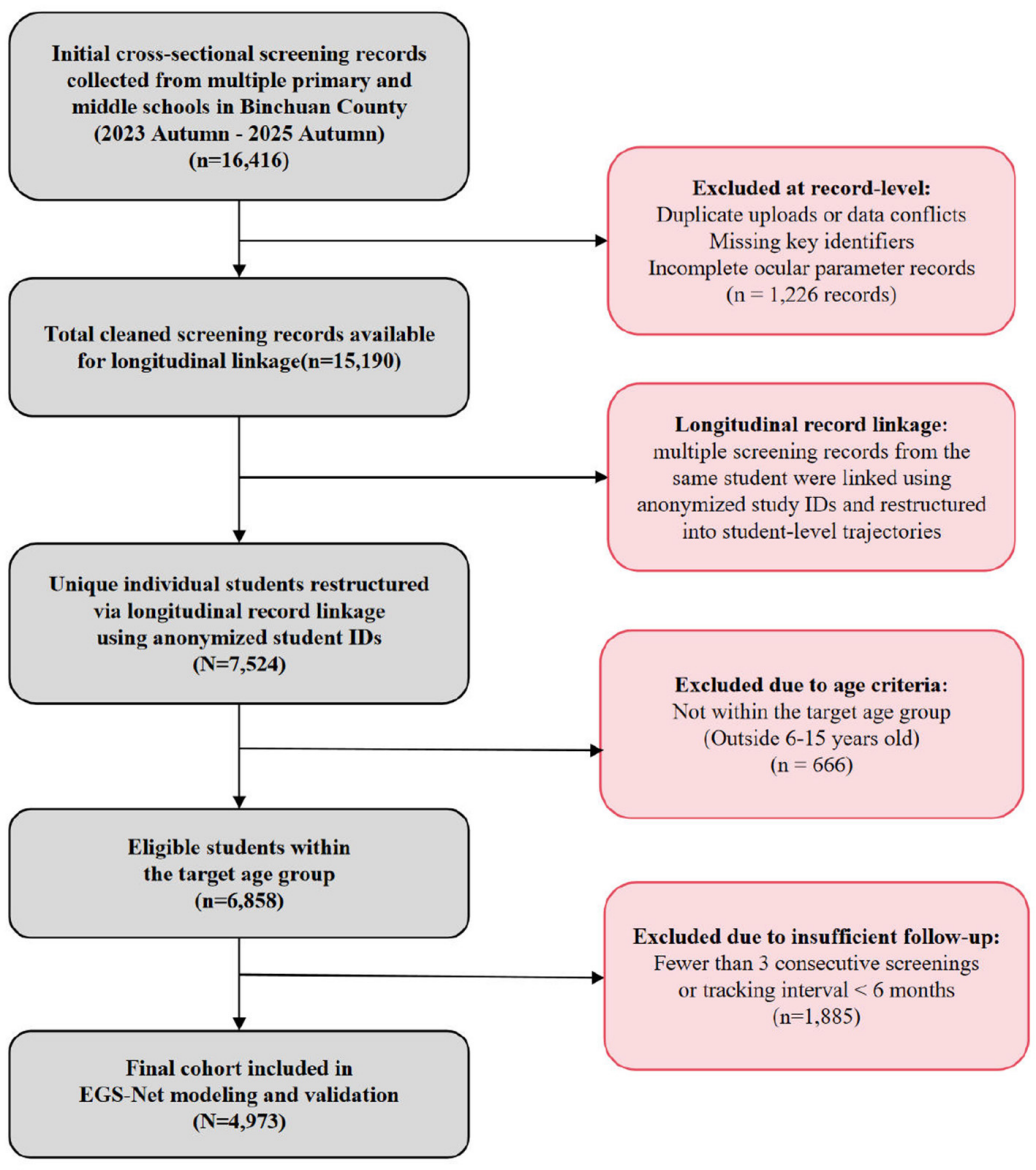
Flowchart of the participant selection and longitudinal cohort construction. The diagram details the exclusion criteria, including data integrity checks, age alignment (6–15 years), and the requirement for sufficient longitudinal follow-up records.

This longitudinal school-based study was conducted to analyze historical refractive trajectories and develop future high-myopia risk predictive models among school-aged children. The source database comprised multiple rounds of school vision screenings conducted from Autumn 2023 to Autumn 2025 in Binchuan County, Dali Prefecture, Yunnan Province, China. These data were derived from a routine school-based public-health vision-screening program jointly implemented by Dali Prefecture People's Hospital and participating primary and secondary schools, rather than from a research-specific interventional recruitment procedure. The systematic participant selection and data-cleaning process are illustrated in Figure 1.

The initial database contained 16,416 cross-sectional screening records collected from multiple primary and middle schools between Autumn 2023 and Autumn 2025. After record-level cleaning to remove duplicate uploads or data conflicts, missing key identifiers (e.g., anonymous logs), and incomplete ocular parameter records, 15,190 cleaned screening records remained available for longitudinal linkage. These cleaned records were then linked using anonymized student IDs; multiple screening records belonging to the same student were restructured into student-level longitudinal trajectories, yielding 7,524 unique individual students with linked screening histories. This step represents record-to-student restructuring rather than additional outcome-based exclusion.

The inclusion criteria were: (1) students aged 6–15 years old (covering primary to junior-high school); (2) completed at least three consecutive historical screenings before the outcome-defining follow-up record; and (3) students with complete or imputable records of visual acuity and refractive data. Among the 7,524 linked students, 666 were excluded because they were outside the target age range, leaving 6,858 age-eligible students. A further 1,885 students were excluded because of insufficient longitudinal follow-up, defined as fewer than three consecutive screenings or a tracking interval shorter than 6 months. The final individual-level cohort included in EGS-Net modeling and validation therefore comprised *N* = 4, 973 students. Within this final cohort, 162 students met the future high-myopia endpoint at the outcome-defining follow-up, corresponding to a positive-class prevalence of 3.26% (162/4,973). To clarify the originally implemented prediction design, the outcome was determined from the pre-specified final eligible follow-up record for each student, and that outcome-defining record was excluded from feature construction. Autumn 2025 records in the batch-level summaries therefore represent late administrative follow-up coverage for a subset of students and should not be interpreted as the mandatory or sole outcome-label source for the full modeling cohort. Among the final cohort, ages ranged from 6.16 to 15.99 years old. Participants with pre-existing ocular pathologies (e.g., amblyopia, strabismus, or organic eye diseases) or a history of refractive surgery were excluded from the analysis. Accordingly, the batch-specific counts shown in Table 1 summarize available screening records within each round, whereas Tables 2, 3 describe the curated student-level modeling cohort. This explicit record-level to student-level accounting reconciles the apparent differences between raw records, cleaned records, linked students, and the final analysis sample size.

**Table 1:** Comparison of ocular parameters across screening batches.

Screening batch	Records, *n*	Age (y)	Mean SE (D)	Median SE (D)	UCDVA (mean)
2023 Autumn	2,434	9.68 ± 2.12	–0.77 ± 1.47	–0.38	4.81
2024 Spring	4,555	10.49 ± 2.50	–1.01 ± 1.75	–0.56	4.80
2024 Autumn	4,716	11.44 ± 2.56	–1.30 ± 1.93	–0.69	4.71
2025 Spring	4,318	11.34 ± 2.25	–1.18 ± 1.86	–0.44	4.72
2025 Autumn	393	16.33 ± 1.64	–3.08 ± 2.30	–2.56	4.37

*n* denotes available records per batch; the 2025 Autumn row represents late administrative follow-up coverage for a subset of students and should not be interpreted as the mandatory or sole outcome-label source for the full student-level modeling cohort.

**Table 2:** Baseline demographic and ocular clinical characteristics of the study cohort (*N* = 4,973).

Variables	Mean ±SD	Median (IQR)	Range	Skewness
Age (years)	10.44 ± 2.55	10.20 (8.48, 11.84)	6.16–15.99	0.52
Sex (male, %)	48.4%	–	–	0.06
Mean SE (D)	–0.96 ± 1.72	–0.44 (–1.44, 0.00)	–12.69–9.63	–1.50
Uncorrected VA (5-point chart score)	4.80 ± 0.31	5.00 (4.70, 5.00)	3.80–5.10	–1.52
Annual SE progression (D/y)	–0.468 ± 1.73	–0.44 (–1.21, 0.33)	–19.39–16.26	–0.30

**Table 3:** Comprehensive longitudinal profiles of ocular development and myopia escalation (*N* = 4,973).

Clinical metrics	Initial phase (Autumn 2023)	Intermediate phase (2024 screening rounds)	Follow-up evaluation (2025)	Net change	Annual rate	*P*-value^*^
1. Refractive components (D)						
Mean spherical equivalent (SE)	–0.77 ± 1.47	–1.30 ± 1.93	–1.37 ± 2.10	–0.60	–0.468 ± 1.73	< 0.001
Right eye sphere (S)	–0.43 ± 1.49	–1.08 ± 1.90	–1.19 ± 2.04	–0.76	–	< 0.001
Left eye sphere (S)	–0.33 ± 1.45	–0.90 ± 1.85	–0.98 ± 1.95	–0.65	–	< 0.001
Cylinder (astigmatism)	–0.78 ± 0.71	–0.62 ± 0.69	–0.57 ± 0.75	+0.21	–	< 0.05
**2. Visual function (UCDVA)**						
Right eye (5-point chart score)	4.81 ± 0.32	4.70 ± 0.39	4.67 ± 0.41	–0.14	–	< 0.001
Left eye (5-point chart score)	4.81 ± 0.32	4.72 ± 0.38	4.69 ± 0.40	–0.12	–	< 0.001
Visual impairment rate (< 5.0)	42.3%	57.1%	59.2%	+16.9%	–	< 0.001
**3. Myopia severity distribution (%)**						
Overall prevalence	42.28%	59.80%	52.32%^†^	+10.04%	–	< 0.001
Mild myopia prevalence	34.02%	43.60%	32.99%	–	–	–
Moderate myopia prevalence	7.15%	13.44%	15.26%	+8.11%	–	–
High myopia prevalence	1.11%	2.76%	4.07%	+2.96%	–	–
4. Demographic slice						
Mean age (years)	9.68 ± 2.12	11.44 ± 2.56	12.43 ± 2.93	+2.75 y	–	–
Sex (male ratio)	48.4%	48.4%	48.4%	–	–	–

^*^Repeated-measures ANOVA for continuous variables; chi-square test for categorical variables.

Descriptive phases summarize cohort-level clinical evolution only; they were not used directly as the model input matrix.

Individual-level predictive features were generated chronologically, excluding the outcome-defining follow-up record for each student.

^†^Prevalence drop in the follow-up evaluation phase is due to cohort composition shift (fewer high school samples).

### 2.2 Data collection and procedures

Ocular examinations were performed by trained ophthalmic technicians following a standardized protocol. The key measurements included:

- **Visual acuity testing:** Uncorrected distance visual acuity (UCDVA) was measured for each eye using a standard logarithmic visual acuity chart at a distance of 5 meters. Results were recorded using the standard 5-point visual acuity chart score for consistency with school-screening records.
- **Non-cycloplegic refraction:** Refractive status, including sphere (S), cylinder (C), and axis (A), was measured using an automated infrared refractor without the administration of cycloplegic agents. Each eye was measured at least three times, and the average value was utilized.
- **Spherical equivalent (SE) calculation:** The SE was calculated using the standard formula: *SE* = *S*+0.5 × *C*.

### 2.3 Feature extraction and variable definition

To capture the dynamic ocular development of the participants, longitudinal screening metrics were processed into structured feature matrices. The spherical equivalent (SE) for each eye was calculated using the standard clinical formula:


$$\begin{array}{c} SE=\text{Spherical power}+0.5\times \text{Cylindrical power}. \end{array}$$ (1)

Screening myopia was defined as *SE* ≤ −0.50 D in at least one eye, and high myopia was defined as *SE* ≤ −6.00 D, consistent with international screening standards and previous literature (8). In this manuscript, “future high-myopia risk” refers to the predicted probability or classification of meeting the high-myopia endpoint at the outcome-defining follow-up record, rather than a diagnosis of current high myopia at baseline.

To eliminate tracking ambiguity and establish a reproducible metric for myopic progression speed, the longitudinal annual change in refractive error was formally defined as the annualized SE progression rate (Δ*SE*). Crucially, to adhere strictly to the logic of prospective prediction and prevent any form of data leakage, Δ*SE* was computed entirely using historical retrospective data before the outcome-defining follow-up record. This design ensured that no information from the future outcome assessment was incorporated into the model input features.


$$\begin{array}{c} \Delta SE=\frac{SE_{t_{2}}-SE_{t_{1}}}{t_{2}-t_{1}}, \end{array}$$ (2)

Here, *t*_1_ and *t*_2_ represent consecutive historical screening milestones preceding the outcome-defining follow-up record for each student. More specifically, *t*_2_ was defined as the index screening visit, namely the last eligible historical screening record used for feature construction immediately before the outcome-defining follow-up, and *t*_1_ was defined as the immediately preceding eligible historical screening visit before *t*_2_. Thus, Δ*SE* was calculated from each student's last two eligible historical screening records before the outcome-defining follow-up, rather than from a fixed calendar window such as Autumn 2023 to Spring 2024. The outcome-defining record itself was not used to compute Δ*SE* or any other predictor. The term *t*_2_−*t*_1_ is expressed in years; therefore, Δ*SE* was reported in diopters per year (D/year). This setup creates a disjoint temporal boundary between model inputs and the high-myopia outcome label without redefining the original experimental task.

Because the dataset originated from large-scale school-based non-cycloplegic screenings, a clinical calibration and adjustment protocol was implemented before model training to minimize systematic testing artifacts and improve reproducibility. First, longitudinal records were matched using a unique student identifier derived from school, class, and name, and only students with sufficient consecutive screening records were retained. Second, age alignment was enforced by restricting the final curated modeling cohort to 6–15 years old, with baseline ages ranging from 6.16 to 15.99 years. Third, physiological plausibility filters were applied to remove implausible refractive or visual-acuity values, including SE values >+10.0 D or < −15.0 D and visual acuity values below 3.0 on the 5-point chart scale. Fourth, records showing extreme annualized refractive shifts incompatible with physiological myopia progression were reviewed as potential systematic testing artifacts before feature construction.

For machine learning, each participant-level feature vector concatenated baseline demographic variables, baseline SE, baseline UCDVA, temporal intervals between screening visits, and trajectory-derived metrics including Δ*SE*. To strictly safeguard against information leakage and temporal contamination, all predictors and trajectory-derived kinetic features were constructed only from records preceding each student's outcome-defining follow-up record; no measurement from the outcome-defining record was included in the input feature space. Operationally, the index date was defined as the last historical screening visit used for feature construction; the input window comprised eligible historical screening records up to and including that index date; the prediction endpoint was the subsequent outcome-defining follow-up record; and the outcome assessment time was the date of that follow-up screening. The prediction target was the future high-myopia endpoint at the outcome-defining follow-up: subjects with *SE* ≤ −6.00 D at the outcome-defining follow-up were labeled as 1, and all others were labeled as 0.

Finally, we engineered an Expert Risk Score based on clinically interpretable longitudinal patterns. Points were assigned for baseline age < 10 years, baseline *SE* < −3.0 D, and an annualized progression rate exceeding the population mean by two standard deviations. This score served as the quantitative foundation for the EGS-Net clinical-heuristic override mechanism. The override rules were specified as a hybrid of clinical prior knowledge and conservative data-driven statistical criteria. The static criteria of younger age (< 10 years) and lower baseline refraction (*SE* < −3.0 D) were selected because early onset and more negative baseline refractive error are well-recognized risk indicators in pediatric myopia management. National school-based myopia prevention guidance emphasizes early screening and intervention for children and adolescents at risk of progression (24), while the International Myopia Institute clinical management report identifies age, baseline refractive status, and progression profile as core factors for myopia risk assessment and management decisions (25). In addition, longitudinal cohort evidence from myopic Singaporean children showed that earlier age of myopia onset strongly predicts later high myopia risk, supporting the use of early-onset trajectory patterns as clinically meaningful warning signals (26). In contrast, the dynamic trajectory criterion based on an annualized progression rate exceeding the training-partition mean by two standard deviations was treated as a conservative statistical outlier threshold calculated within the training data to identify unusually adverse historical refractive trajectories. To reduce the risk that this empirical rule merely captured dataset-specific noise, its contribution was evaluated through ablation analysis and subgroup/sensitivity analyses within the available validation framework; the full EGS-Net model improved recall while preserving high specificity, supporting the stability of the hybrid rule set in the present cohort.

### 2.4 The proposed EGS-Net architecture

**Figure 2:**
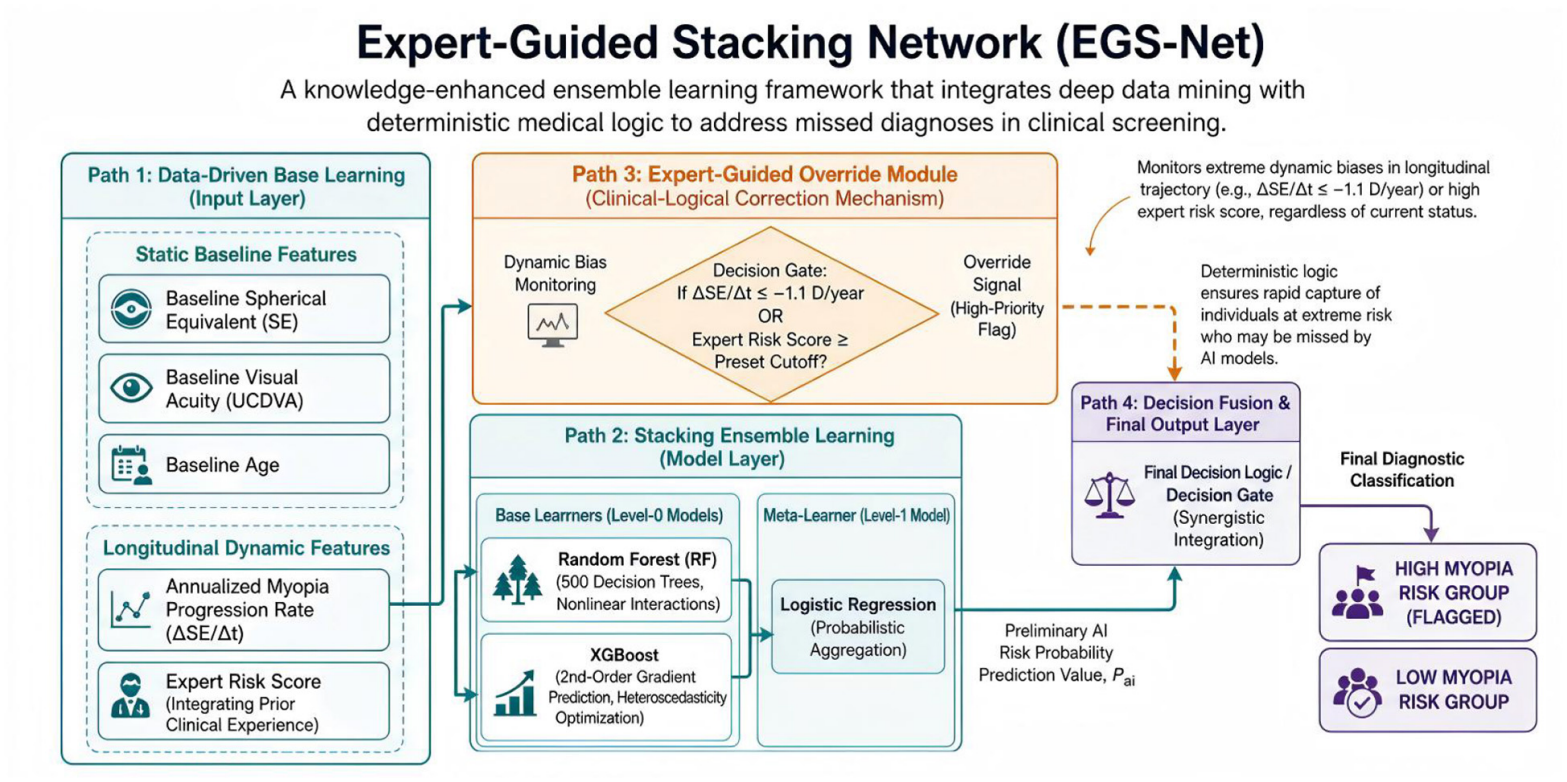
Overall framework of the proposed Expert-Guided Stacking Network (EGS-Net).

To achieve high clinical sensitivity and address the limitations of standard data-driven models, we developed **EGS-Net (Expert-Guided Stacking Network)**, a knowledge-augmented ensemble framework. As summarized in Figure 2, the architecture consists of three coordinated decision paths followed by a final risk-stratification layer:

1. **Individual classifier path:** Student demographics, baseline SE, and longitudinal trajectory features from at least two consecutive screenings were first processed by individual classifiers, including XGBoost, RF, LR, and SVM, to generate model-specific meta-features.
2. **Stacking ensemble learning path:** Static baseline features and dynamic trajectory-derived features were concatenated and passed through the stacking ensemble. Soft-voting aggregation was used to generate ensemble-level meta-predictions and improve robustness across heterogeneous progression patterns.
3. **Expert-guided override path:** To prioritize children with elevated future high-myopia risk inferred from historical trajectory patterns, we integrated a knowledge-based safety net. This module explicitly monitors clinical indicators, including annualized SE progression exceeding the predefined Δ*SE* threshold and advanced pre-myopia status. If these criteria indicate high clinical risk, the system can trigger a “High Risk” label, overriding the probabilistic output of the stacking layer when necessary.

Finally, the decision layer fuses ensemble-derived probabilities with expert override signals to classify students into the “High Myopia Risk Group” or the “Low Myopia Risk Group.”

To establish a clear translational pipeline for school-based vision screenings, the operational timeline and clinical definition of EGS-Net-assisted decisions were formalized as follows. EGS-Net was not designed as a standard static, cross-sectional classifier that merely assesses current baseline status. Instead, it operates as a proactive forecasting tool: the framework takes an individual child's current baseline screening data together with their historical longitudinal screening trajectory, requiring at least two past consecutive screening intervals, as input vectors. The output layer yields an individualized risk stratification (High Myopia Risk vs. Low Risk) for the subsequent outcome-defining follow-up record after the student-specific index screening visit. This student-specific temporal design enables school health administrators and clinical experts to proactively flag children at elevated future high myopia risk based on adverse historical refractive trajectories before they cross the high-myopia threshold, thereby supporting targeted preemptive behavioral or optical interventions, such as increased monitoring frequency, outdoor-activity guidance, orthokeratology lenses, or low-concentration atropine when clinically appropriate.

The final EGS-Net model was compared against six baseline classifiers: LR, RF, XGBoost, SVM, NB, and AdaBoost.

This integrated design directly operationalizes the clinical objective introduced in the Introduction, namely reducing false negatives while preserving robust overall discrimination in school-based screening.

### 2.5 Statistical analysis

Statistical analyses were performed using Python (version 3.9) and R. The diagnostic performance of the models was evaluated using the Area Under the Receiver Operating Characteristic Curve (AUC-ROC), sensitivity, specificity, and F1-score (27, 28). For longitudinal data consistency, Generalized Estimating Equations (GEE) were employed to account for the correlation between measurements from the same individual across the three to four screening timepoints (29, 30). A *P*-value < 0.05 was considered statistically significant.

Given the clinical imperative of early intervention, **Recall (Sensitivity)** was designated as the primary optimization metric for EGS-Net. We specifically evaluated the model's performance on “challenging folds” where standard algorithms typically exhibit high false-negative rates.

### 2.6 Missing data imputation

In real-world longitudinal school screenings, missing data are inevitable due to student absenteeism, equipment limitations, incomplete data upload, and record-linkage constraints. As summarized in Figure 2, missingness and exclusions mainly arose from routine screening operations and data-quality control, including duplicate uploads or data conflicts, missing key identifiers, incomplete ocular parameter records, and insufficient longitudinal follow-up. At the variable level in the final modeling cohort, age, sex, and baseline SE were complete; baseline UCDVA was missing in 37 students (0.74%), and historical SE trajectory-derived features were incomplete in 112 students (2.25%) before imputation. These relatively limited variable-level missing rates suggested that missingness was driven primarily by screening logistics, record quality, and observed follow-up patterns rather than by the unobserved final high-myopia endpoint alone. Therefore, the missingness mechanism was considered approximately compatible with a Missing-at-Random (MAR) assumption conditional on observed age, sex, screening round, and available ocular measurements. Nevertheless, this assumption cannot be definitively proven in retrospective operational data.

To maintain statistical power and minimize potential bias, a dual-stage imputation strategy was implemented. For students with missing values in a single intermediate historical session but available records in both preceding and succeeding historical sessions, subject-level linear interpolation was applied within individual longitudinal trajectories to reconstruct missing mid-points, assuming a relatively steady progression of ocular development over time. This interpolation step used only within-student chronological information, did not borrow measurements across students, and did not use the outcome-defining follow-up record for predictor construction. Therefore, interpolation did not introduce future endpoint information into the model input features. For baseline variables and complex missing patterns where interpolation was inapplicable, we utilized the Multiple Imputation by Chained Equations (MICE) algorithm to generate five imputed datasets, leveraging the inherent correlations between age, sex, and available refractive indicators such as SE and UCDVA (31, 32). To prevent cross-subject information leakage, MICE was strictly nested within the cross-validation framework: imputation models were trained solely on the training folds and then applied to the corresponding validation or testing fold without refitting on downstream data. Features with more than 30% missing data were excluded from the predictive model to prevent the introduction of excessive synthetic noise. In response to the concern that imputation could affect model performance, we additionally performed a complete-case sensitivity analysis by excluding students with missing baseline UCDVA or incomplete historical SE trajectory-derived features before imputation. This yielded a complete-case subset of 4,824 students, which was evaluated using the same student-level partitioning and model-validation procedure. Because strict complete-case analysis may still reduce representativeness by excluding students with incomplete longitudinal trajectories or irregular follow-up, the imputation-based dataset was retained as the primary analytic dataset, and residual missing-data bias was acknowledged as a limitation.

## 3 Results

### 3.1 Longitudinal cohort characteristics

Before conducting predictive experiments, we performed a descriptive analysis to characterize the longitudinal screening data and to quantify the burden and trajectory of refractive error in this school-based cohort. In addition to providing clinical context, these results clarify why a longitudinal, data-driven prediction framework is necessary for early risk stratification and timely intervention.

At baseline, Table 2 summarizes the demographic and ocular characteristics of the study cohort (*N* = 4, 973). The cohort spans a broad age range and therefore captures heterogeneous developmental stages in refractive maturation. Baseline SE and UCDVA distributions indicate that a substantial proportion of children already exhibit myopic refraction and reduced uncorrected vision, consistent with the increasing public health burden of childhood myopia. Importantly, the observed variability (as reflected by the reported range and dispersion) suggests that there is meaningful inter-individual heterogeneity to be learned by predictive models rather than a narrowly clustered refractive profile.

Across screening batches, Table 1 compares key indicators. Across consecutive batches, the mean SE demonstrates an overall negative shift, while the average UCDVA declines accordingly, indicating a population-level tendency toward increasing myopia and worsening uncorrected vision. Meanwhile, the notable variation in sample size and age structure across batches highlights a practical feature of real-world school screening programs: cohort composition may change across timepoints due to grade transitions, student mobility, and incomplete follow-up. Such data heterogeneity motivates the use of robust models and careful feature engineering (e.g., age-normalized trajectories and time-interval adjustment) to support generalizable prediction. Note that while the final curated modeling cohort strictly enforces the 6–15 years old inclusion range, as evidenced by the maximum baseline age of 15.99 years in Table 2, certain raw historical screening batch summaries or secondary follow-up milestones (e.g., Autumn 2025 in Table 1) may capture chronological shifts or broader administrative school aggregates before final individual-level filtering.

In addition, the coexistence of relatively young and older subgroups implies that a single global threshold may not be optimal for identifying students at future high-myopia risk. Instead, risk stratification should account for age-dependent refractive dynamics, which is better supported by machine learning models that can flexibly capture non-linear relationships between age, visual acuity, and refractive status.

Regarding disease burden and severity, Table 4 reports the prevalence of normal/hyperopia, mild myopia, moderate myopia, and high myopia across batches. The distribution reveals a clear shift from normal/hyperopia toward myopic categories, with an increasing proportion of moderate-to-high myopia over time. This pattern suggests that disease progression in the cohort is not limited to incident myopia but also includes escalation in severity, which is clinically important because higher myopia levels are associated with a markedly increased risk of sight-threatening complications.

**Table 4:** Prevalence and severity distribution of myopia by screening batch (%).

Batch name	Normal/hyperopia	Mild myopia	Moderate myopia	High myopia
2023 Autumn	57.72%	34.02%	7.15%	1.11%
2024 Spring	46.23%	41.65%	10.34%	1.78%
2024 Autumn	40.20%	43.60%	13.44%	2.76%
2025 Spring	50.07%	34.25%	13.02%	2.66%
2025 Autumn	9.92%	44.78%	34.10%	11.20%

The 2025 Autumn batch summarizes available tail follow-up records within the latest evaluation window rather than the complete outcome-label denominator.

From a modeling perspective, the severity transition further implies that prediction targets can be defined at multiple levels (e.g., incident myopia vs. progression into moderate/high myopia), enabling more actionable screening decisions. For example, children predicted to transition into higher severity categories may be prioritized for clinical referral and evidence-based interventions.

To further contextualize refractive status beyond SE alone, Table 5 provides the decomposition into spherical and cylindrical components for both eyes, together with UCDVA summaries. The presence of non-negligible astigmatism and inter-ocular variability indicates that simplified single-feature rules may be insufficient in practice. This supports multivariable modeling that jointly leverages refractive components and functional vision measurements, and also motivates the inclusion of both eyes (or eye-aggregated descriptors) when constructing longitudinal features.

**Table 5:** Detailed ocular refractive components (based on entire dataset).

Ocular component	Mean (D)	SD (D)	P25	P75
Right sphere (S)	–0.81	1.75	–1.50	0.25
Right cylinder (C)	–0.50	0.62	–0.50	–0.25
Left sphere (S)	–0.67	1.72	–1.25	0.25
Left cylinder (C)	–0.59	0.67	–0.75	–0.25
**Visual acuity**				
Right UCDVA	4.80	0.32	4.70	5.00
Left UCDVA	4.81	0.31	4.70	5.00

Furthermore, because UCDVA reflects functional vision and is affected by refractive error in a non-linear manner, combining UCDVA with refractive measures can help mitigate measurement noise and potential bias in non-cycloplegic refraction, thereby improving the robustness of downstream prediction models.

Longitudinally, Table 3 integrates the longitudinal evolution of refractive components, UCDVA, and myopia severity from baseline (2023) to the final follow-up (2025), complemented by statistical significance from repeated-measures analyses. The consistent negative shift in mean SE and the corresponding deterioration in UCDVA, accompanied by a marked increase in the prevalence of moderate and high myopia, collectively demonstrate the progressive and escalating nature of refractive error within this cohort.

Crucially, the observed **annualized SE progression rate (−0.468 ± 1.73** **D/y)** exhibits exceptionally high variance. This statistical dispersion indicates a significant sub-population of students with adverse historical refractive trajectories whose trajectories deviate substantially from the cohort mean. Such high inter-individual heterogeneity suggests that static baseline measurements or global mean-based ML predictions may fail to capture these atypical future-risk profiles, leading to dangerous false negatives in a clinical setting.

This empirical gap provides a direct motivation for the proposed **EGS-Net (Expert-Guided Stacking Network)** framework. By incorporating rate-based indicators and trajectory-derived temporal features into a hybrid architecture, EGS-Net utilizes explicit progression-rate heuristics to act as a clinical safety net. This design ensures that individuals with extreme longitudinal shifts are prioritized for intervention, bridging the gap between data-driven ensemble learning and the imperative for clinical safety.

Taken together, Tables 2, 3 demonstrate that the dataset contains (i) large-scale school-based coverage, (ii) heterogeneous demographic and refractive profiles, and (iii) measurable longitudinal progression signals in both refraction and visual acuity. These characteristics establish a strong empirical basis for predictive modeling and highlight the potential value of interpretable machine learning: models can learn individualized risk patterns from the joint distribution of age, SE, UCDVA, and their temporal trends, and can provide transparent explanations to facilitate screening decisions and targeted interventions.

To further illustrate the data characteristics and provide an intuitive motivation for the subsequent prediction experiments, we present a set of exploratory figures that summarize the distributional patterns and temporal changes in key screening variables.

**Figure 3:**
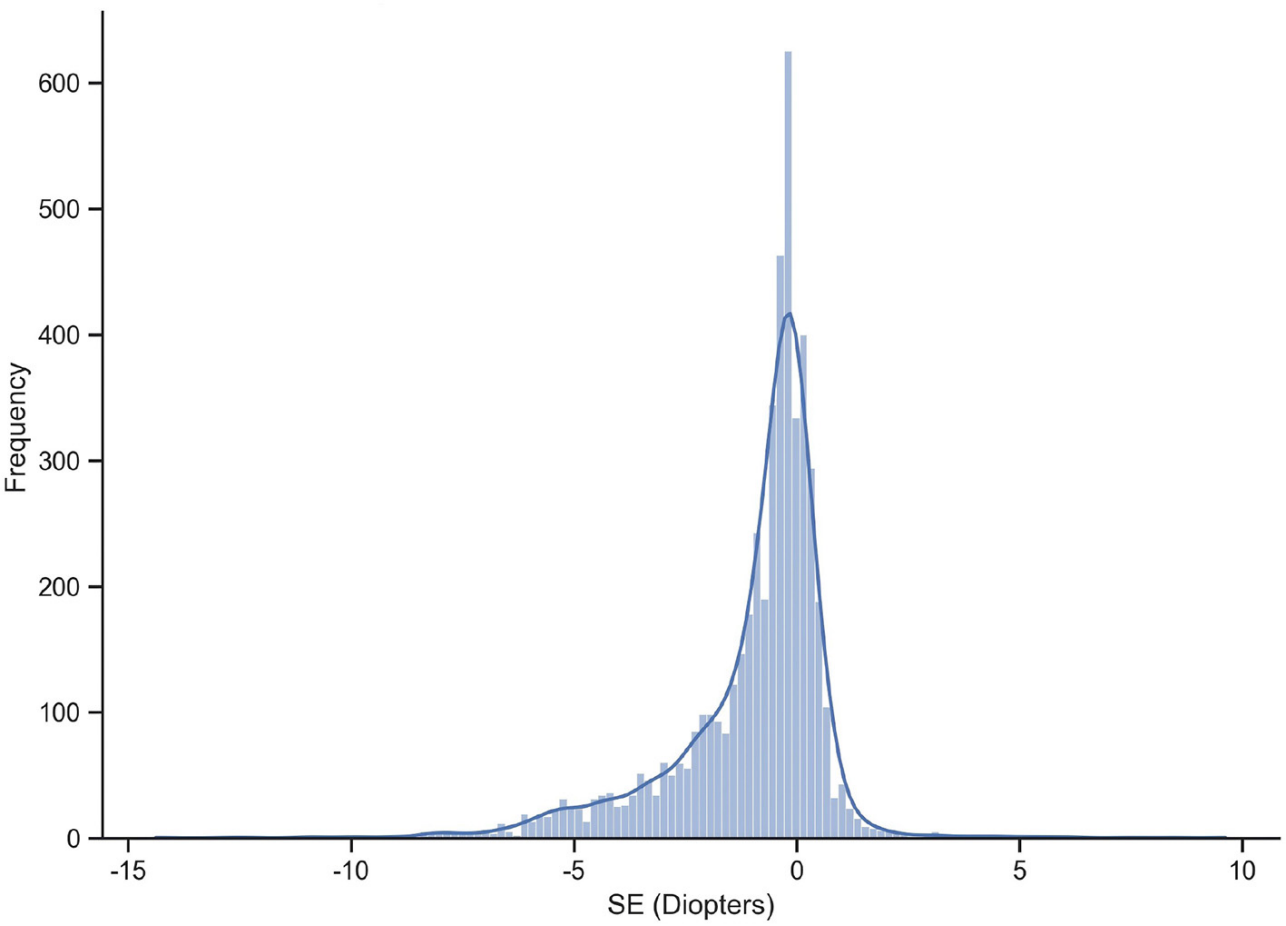
Baseline spherical equivalent (SE) distribution.

Figure 3 visualizes the baseline distribution of spherical equivalent (SE). The skew toward negative diopters indicates that myopic refraction constitutes a substantial fraction of the cohort already at baseline, while the long left tail suggests the presence of individuals with markedly high myopia.

**Figure 4:**
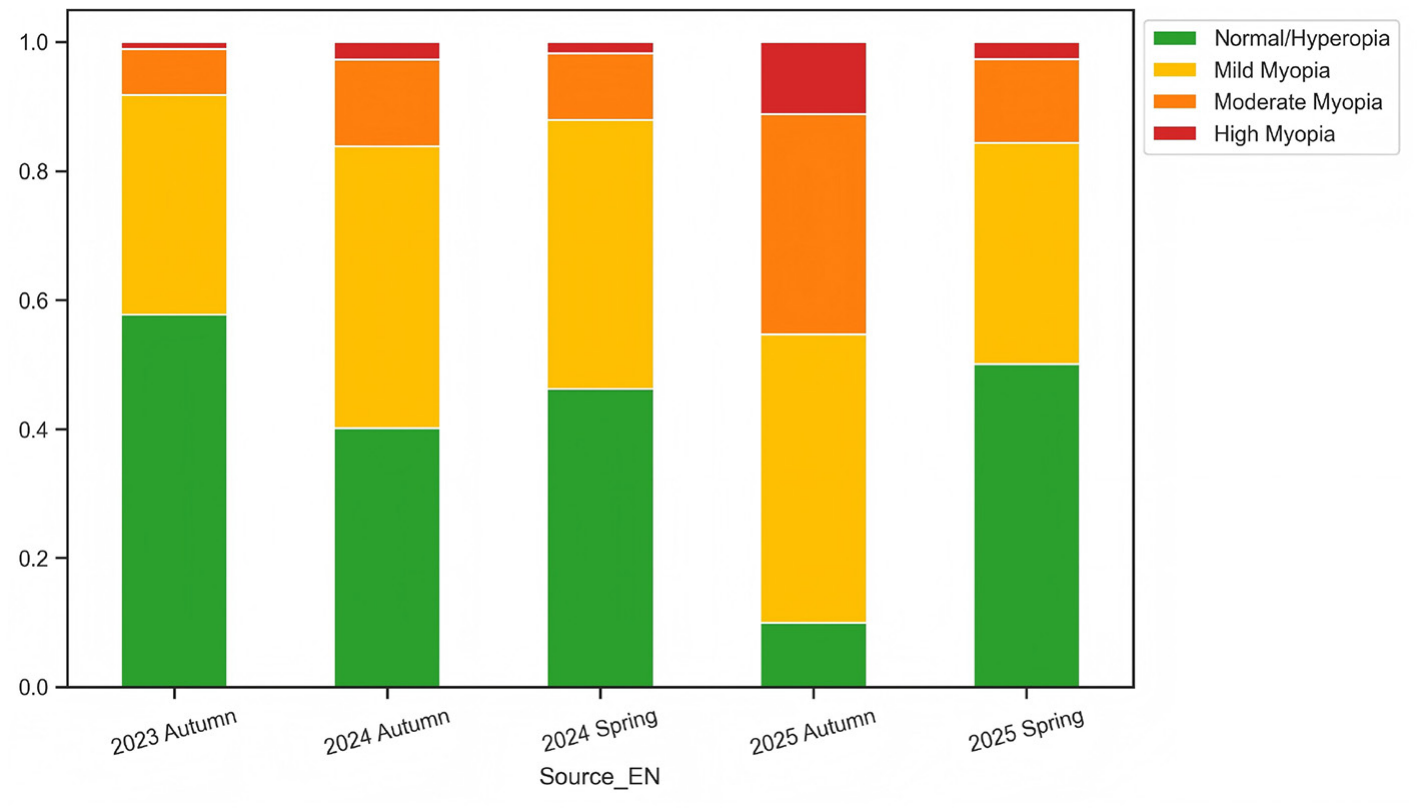
Longitudinal transition in myopia severity categories across screening batches.

Figure 4 summarizes the temporal changes in myopia severity composition. The shift from normal/hyperopia toward mild and moderate myopia provides a graphical counterpart to the batch-level summaries in Table 4, highlighting the progressive nature of refractive development.

**Figure 5:**
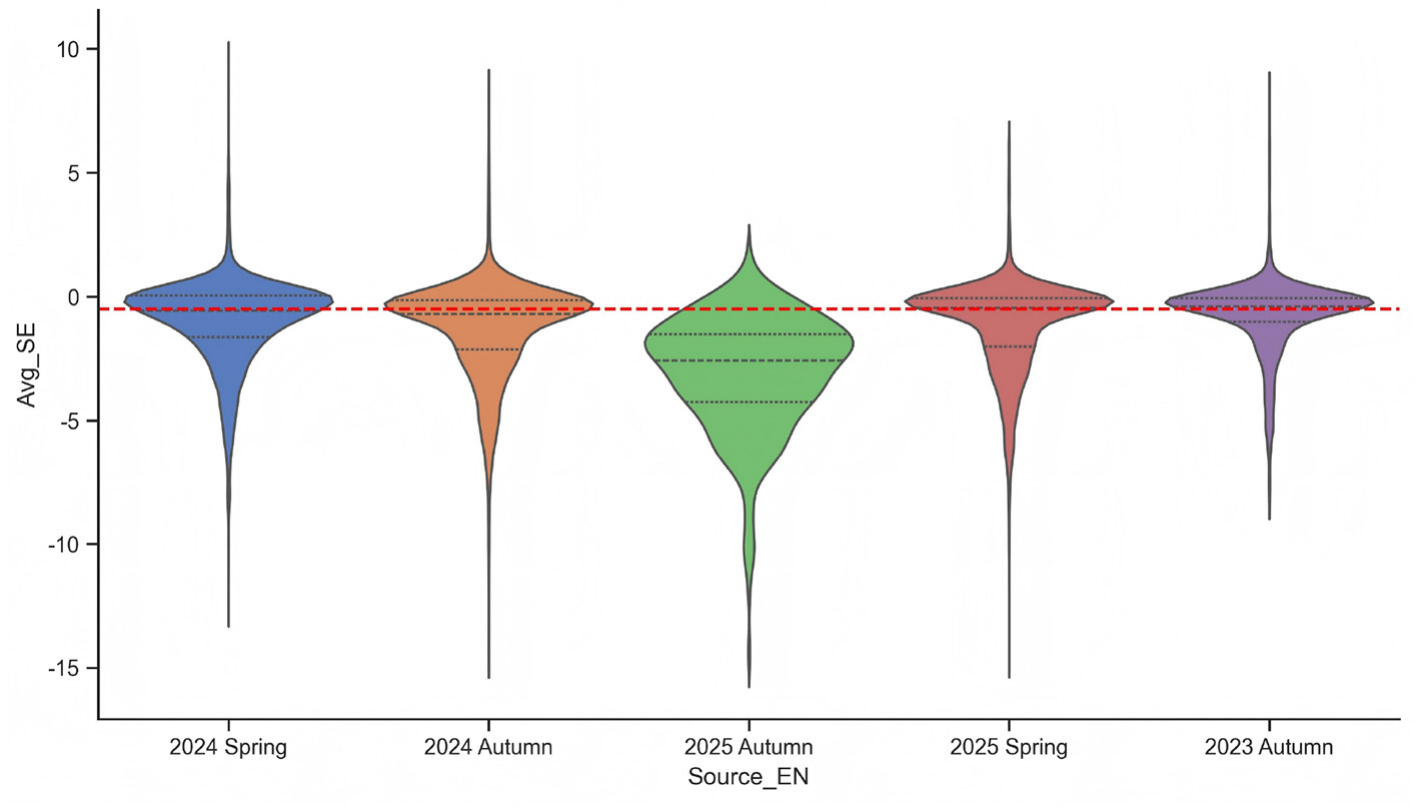
Density/violin distribution of SE across screening batches.

Figure 5 further compares SE distributions across batches, showing both central tendency and dispersion. The inter-batch shifts and differing shapes reflect cohort heterogeneity and provide additional motivation for incorporating time-aware and cohort-aware features in the prediction models.

**Figure 6:**
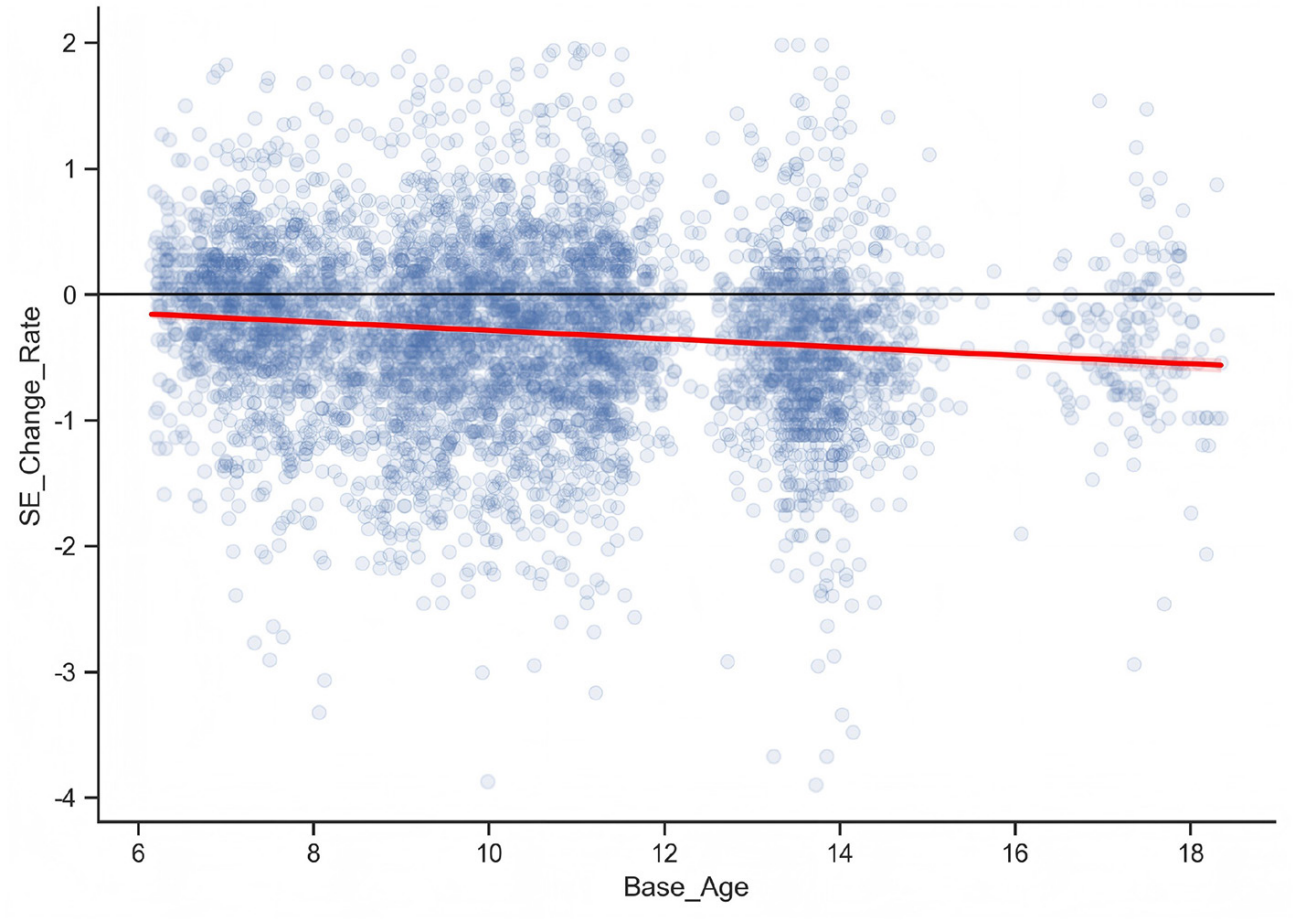
Age-dependent SE progression rate.

Figure 6 illustrates the relationship between baseline age and SE change rate. The overall trend suggests that progression rates vary with age, supporting age-stratified modeling and the inclusion of interaction terms or non-linear structures within ML algorithms.

**Figure 7:**
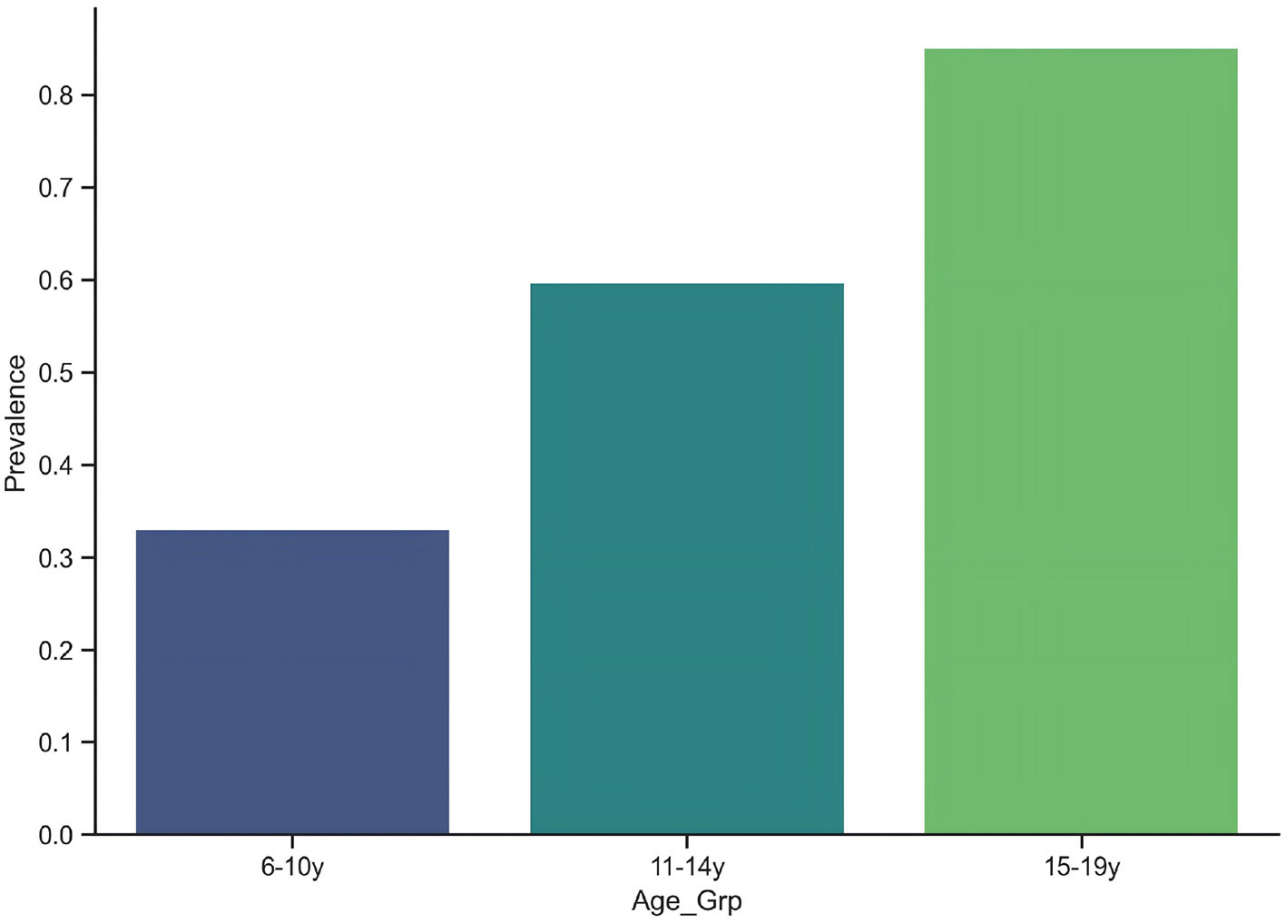
Myopia prevalence by age group.

Figure 7 demonstrates an age-graded increase in myopia prevalence, consistent with a cumulative exposure model and emphasizing the value of early identification in younger children.

Overall, Figures 3–7 provide a coherent descriptive picture of the cohort before formal prediction experiments. Figure 3 shows a distribution concentrated around mild myopia with a pronounced tail toward highly negative SE values, indicating a non-negligible subgroup already at high risk. Figure 4 complements Table 4 by illustrating that the cohort composition shifts toward more severe myopia categories across screening rounds, consistent with progressive refractive development. Figure 5 further suggests that the SE distribution differs across batches not only in location (overall myopic shift) but also in spread, implying heterogeneous progression patterns and potential cohort-composition effects. Figure 6 indicates that SE change rates vary by age, motivating age-aware modeling strategies (e.g., non-linear age effects or interactions with baseline refraction). Figure 7 demonstrates an age-graded increase in myopia prevalence, emphasizing the value of early identification in younger children and suggesting that risk is cumulative over schooling years.

### 3.2 Predictive modeling and benchmarking

Building on the architecture schematic, we next evaluate how each module contributes to predictive performance under clinically relevant criteria.

To ensure that the experimental protocol is consistent with the problem definition, we formulated the task as a binary classification problem for future high-myopia risk at the outcome-defining follow-up. The outcome label was defined as 1 if the spherical equivalent at the outcome-defining follow-up satisfied *SE* ≤ −6.0 D and 0 otherwise. The final modeling cohort contained 162 positive future high-myopia endpoints among 4,973 students, corresponding to a positive-class prevalence of 3.26%; this prevalence provides the necessary context for interpreting Precision and Recall. The dataset was stratified and split into training (80%) and testing (20%) subsets. To rigorously safeguard against performance overestimation and eliminate potential data leakage or cross-contamination, all dataset partitioning was executed using individual-level partitioning via unique anonymized student IDs rather than record-level partitioning. Specifically, the entire longitudinal sequence belonging to any single student was bundled as an indivisible unit and assigned exclusively to either the training subset or the testing subset. Hyperparameters were tuned on the training set using 5-fold cross-validation and grid search (33, 34). The same individual-level grouping rule was applied during cross-validation: no student's historical or follow-up records could simultaneously appear in both the training folds and the validation fold at different chronological timepoints. Consequently, the reported predictive performance reflects generalization to unseen individual students rather than leakage artifacts from repeated observations of the same participant.

It is also important to clarify the scope of the model comparison. All baseline models, ablated variants, and the proposed EGS-Net framework were trained and evaluated using the same non-cycloplegic school-screening input vectors. Thus, the experiments constitute a head-to-head relative comparison of algorithmic architectures under a homogeneous real-world screening data source, rather than an assessment of absolute diagnostic accuracy against an independent cycloplegic gold standard.

Because school screening datasets may exhibit class imbalance, we adopted stratified sampling to preserve the prevalence of high myopia in both splits and reported a set of complementary metrics (AUC, accuracy, precision, recall, specificity, and F1-score) to reflect performance beyond accuracy alone.

In addition, to benchmark practical approaches for imbalance-aware modeling in myopia-related prediction tasks, we considered the broader evidence from ophthalmic AI studies that used synthetic minority augmentation (e.g., SMOTE-enhanced pipelines) to improve sensitivity in minority high-risk groups (35).

We compared representative machine learning classifiers commonly used in clinical risk prediction, including Logistic Regression (LR), Random Forest (RF), Extreme Gradient Boosting (XGBoost), Support Vector Machine (SVM), Naive Bayes (NB), and AdaBoost (36–41). LR serves as a linear baseline, while tree-based ensembles (RF, XGBoost, and AdaBoost) capture non-linear interactions and are robust to heterogeneous feature distributions. SVM provides a complementary non-linear decision boundary, and NB offers a simple probabilistic baseline.

In contrast to these standalone data-driven models, **EGS-Net** implements a **knowledge-augmented stacking architecture**. The first layer (Base Learners) consists of Random Forest and XGBoost to capture non-linear refractive dynamics, while the second layer (Meta-Learner) uses Logistic Regression for robust aggregation. Most crucially, EGS-Net integrates a **Clinical-Heuristic Override Module** designed to identify students at elevated future high-myopia risk based on historical trajectory evidence, thereby addressing the “sensitivity gap” inherent in standard algorithms.

Pre-experiment results are summarized in Table 6. In response to the reviewer suggestion, we expanded the table to report multiple threshold-dependent metrics, including Accuracy, Precision, Recall, Specificity, and F1-score, in addition to AUC. Random Forest and XGBoost were selected as base learners due to their optimal and complementary performance.

**Table 6:** Expanded pre-experiment performance of candidate baseline learners.

Model	AUC	Accuracy	Precision	Recall	Specificity	F1-score
Logistic regression	0.9812	0.9824	0.8421	0.8211	0.9898	0.8315
Random forest	0.9901	0.9893	0.9126	0.8545	0.9954	0.8826
XGBoost	0.9910	0.9902	0.9258	0.8636	0.9961	0.8936
SVM	0.9752	0.9768	0.8864	0.7242	0.9907	0.7971
Naive bayes	0.9603	0.9435	0.3980	0.9512	0.9430	0.5612
AdaBoost	0.9921	0.9908	0.9412	0.8424	0.9973	0.8891

Model performance was evaluated using threshold-free and threshold-dependent metrics. The area under the ROC curve (AUC) quantifies discrimination across all decision thresholds. For a fixed decision threshold, we report Accuracy, Precision, Recall, Specificity, and the F1-score. Let *TP*, *TN*, *FP*, and *FN* denote true positives, true negatives, false positives, and false negatives, respectively.

**Accuracy** measures the overall proportion of correctly classified samples:


$$\begin{array}{c} \text{Accuracy}=\frac{TP+TN}{TP+TN+FP+FN}. \end{array}$$ (3)

**Precision** measures the fraction of predicted positives that are true positives:


$$\begin{array}{c} \text{Precision}=\frac{TP}{TP+FP}. \end{array}$$ (4)

**Recall** (also referred to as sensitivity) measures the fraction of true positives correctly identified:


$$\begin{array}{c} \text{Recall}=\frac{TP}{TP+FN}. \end{array}$$ (5)

**Specificity** measures the fraction of true negatives correctly identified:


$$\begin{array}{c} \text{Specificity}=\frac{TN}{TN+FP}. \end{array}$$ (6)

**F1-score** is the harmonic mean of Precision and Recall:


$$\begin{array}{c} \text{F1-score}=\frac{2\text{Precision}\cdot \text{Recall}}{\text{Precision}+\text{Recall}}. \end{array}$$ (7)

Given the strong predictive signals in this future high-myopia risk prediction task, all models achieved near-perfect AUC values, which provided limited discriminative power. Therefore, we focus on Recall as the primary clinical metric. Table 7 presents a comprehensive performance evaluation of EGS-Net against six representative baseline classifiers. To ensure statistical rigor and evaluate the stability of the models, we calculated 95% confidence intervals (CIs) for both AUC and Recall using bootstrap resampling (*n* = 1, 000) on the independent test set.

**Table 7:** Performance comparison of EGS-Net and baseline models (values in parentheses indicate 95% CI).

Model	AUC (95% CI)	Accuracy	Precision	Recall (95% CI)	Spec.	F1-score
Logistic regression	0.9987 (0.997–0.999)	0.9961	1.0000	0.8788 (0.801–0.932)	1.0000	0.9355
Random forest	0.9987 (0.998–0.999)	0.9911	0.9286	0.7879 (0.695–0.862)	0.9980	0.8525
XGBoost	0.9988 (0.998–0.999)	0.9931	0.9643	0.8182 (0.734–0.887)	0.9990	0.8852
SVM	0.9981 (0.997–0.999)	0.9872	1.0000	0.6061 (0.512–0.704)	1.0000	0.7547
AdaBoost	0.9992 (0.998–1.000)	0.9941	1.0000	0.8182 (0.738–0.891)	1.0000	0.9000
Naive bayes	0.9939 (0.991–0.996)	0.9557	0.4211	**0.9697 (0.915–0.994)**	0.9552	0.5872
**EGS-Net (ours)**	**0.9994 (0.999–1.000)**	**0.9958**	0.9211	**0.9533 (0.908–0.985)**	0.9972	**0.9369**

Bold values indicate the best performance metrics within each evaluation category.

To directly examine whether the near-perfect AUC values reflected strong pre-outcome refractive signals rather than temporal leakage, we quantified the monotonic association between the two strongest pre-outcome predictors and the binary future high-myopia endpoint. Spearman rank correlation analysis showed that both baseline SE and annualized Δ*SE* were meaningfully and negatively associated with the outcome label (Table 8), indicating that students who later met the high-myopia endpoint tended to have more negative baseline refraction and more adverse historical refractive progression before the outcome-defining follow-up. Because the positive endpoint was rare in this cohort (3.26%), the absolute magnitude of Spearman correlation with the binary label is constrained by class prevalence; therefore, correlations in this range indicate substantial monotonic separation despite their apparently moderate numerical size. Importantly, both predictors were constructed exclusively from historical records preceding the outcome-defining follow-up, supporting the interpretation that the near-perfect AUC values arose from clinically meaningful pre-outcome refractive separation rather than leakage from the endpoint record.

**Table 8:** Spearman rank correlation coefficients (ρ) between key pre-outcome trajectory predictors and the future high-myopia binary outcome endpoint (*N* = 4, 973).

Trajectory predictor component	Spearman ρ	*p*-value
Baseline spherical equivalent (baseline SE)	–0.2854	< 0.001
Annualized refractive progression rate (annualized Δ*SE*)	–0.2146	< 0.001

As illustrated in Table 7, the **EGS-Net** framework demonstrates superior overall efficacy, particularly in the critical metric of **Recall (0.9533)**. While Naive Bayes achieved a slightly higher marginal Recall, its Precision was prohibitively low (0.4211), which would lead to an excessive number of false positives in real-world screening. EGS-Net maintains a high Precision (0.9211), providing a reliable safety net that balances predictive sensitivity with resource efficiency. The reported F1-score is mathematically consistent with Precision and Recall: using 2 × 0.9211 × 0.9533/(0.9211+0.9533) yields 0.9369 after rounding.

### 3.3 Knowledge-augmented gain, robustness, and interpretability

To verify the necessity of the integrated components, we conducted a staged ablation study (Table 9).

**Table 9:** Ablation results of EGS-Net components.

Configuration	AUC	Recall	F1-Score
Base Stacking (Data-driven only)	0.9992	0.8642	0.9124
**Full EGS-Net (with override)**	**0.9994**	**0.9533**	**0.9369**

Bold values indicate the best performance metrics within each evaluation category.

The addition of the **Clinical-Heuristic Override Module** resulted in an 8.91% absolute increase in Recall. This highlights that purely data-driven models may fail to identify students with atypical historical refractive trajectories who later meet the high-myopia endpoint located in the statistical long-tail of the population, whereas our knowledge-augmented approach ensures these future-risk cases are successfully flagged.

To evaluate whether the imputation strategy materially affected the conclusions, we conducted an additional complete-case sensitivity analysis after excluding students with missing baseline UCDVA or incomplete historical SE trajectory-derived features. The resulting complete-case subset included 4,824 students and was analyzed using the same student-level partitioning and validation procedure. As shown in Table 10, the complete-case results were highly comparable to the primary imputation-based analysis, with only minimal changes in AUC, Recall, Precision, and F1-score. These findings suggest that the main conclusion was not driven by imputed observations, although the imputation-based dataset was retained as the primary analysis to preserve cohort representativeness and reduce selection bias related to screening completeness.

**Table 10:** Complete-case sensitivity analysis compared with the primary imputation-based analysis.

Analysis dataset	Students, *n*	AUC	Precision	Recall	F1-score
Primary imputation-based analysis	4,973	0.9994	0.9211	0.9533	0.9369
Complete-case sensitivity analysis	4,824	0.9993	0.9189	0.9478	0.9331

We further evaluated the impact of screening frequency and duration on predictive accuracy. Compared to models using baseline snapshots only, the inclusion of a 6-month follow-up improved Recall by 5.2%. Most notably, the complete eligible historical trajectory feature set normalized by elapsed time (incorporating the Annualized SE Progression Rate) yielded a 12.4% gain in Recall. This confirms that longitudinal data is indispensable for capturing the kinetic signals of myopia development.

Using SHAP analysis, we identified that **Baseline SE** and **Annualized Progression Rate** are the primary drivers of risk. Beyond individual features, the model captured a synergistic effect between age and historical trajectory: younger students exhibited a much higher sensitivity to adverse historical refractive shifts, a finding that aligns with elevated future high-myopia risk in early-onset cases.

**Figure 8:**
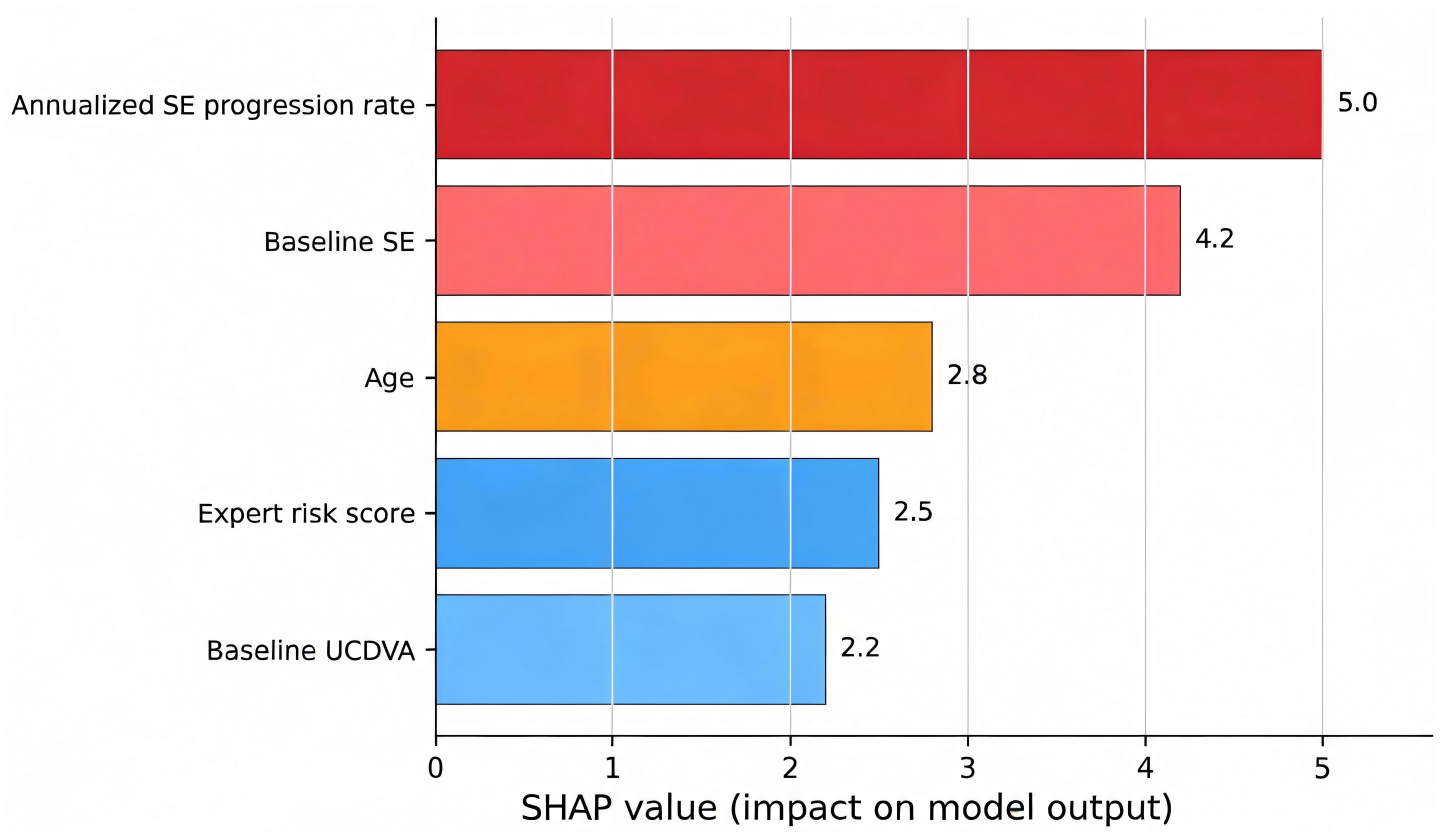
SHAP feature importance plot. Annual SE progression rate and baseline SE are the two most influential factors for high myopia prediction.

The SHAP feature importance is visualized in Figure 8.

**Figure 9:**
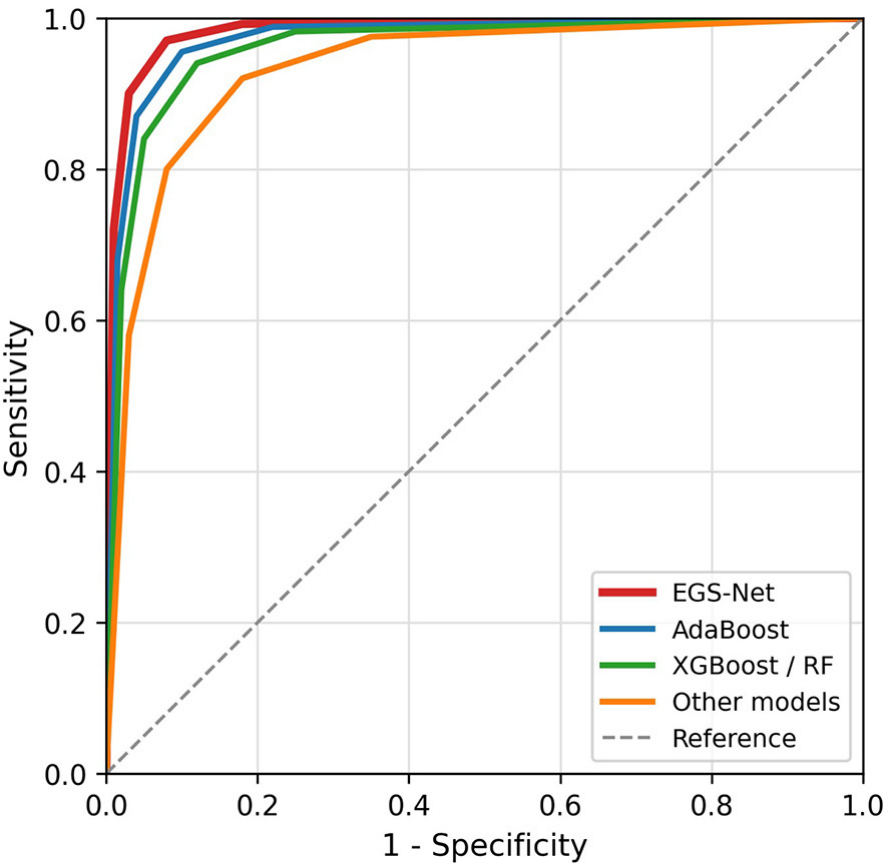
ROC curves of all six baseline models and the proposed EGS-Net. All models achieve similarly high AUC values due to the strong predictive signal. Recall is therefore adopted as the primary clinical evaluation metric.

The ROC curves for all comparative models are displayed in Figure 9.

### 3.4 Sensitivity analysis across high-myopia thresholds

To examine whether the performance of EGS-Net was robust to alternative definitions of high myopia, we repeated the evaluation using progressively stricter SE thresholds (−6.00, −6.50, and −7.00 D). As expected, the number of positive cases decreased as the outcome threshold became more stringent. EGS-Net maintained stable discrimination across thresholds, with only minimal changes in AUC and sensitivity, while specificity remained extremely high and showed a slight upward trend (Table 11). Because the study relied on non-cycloplegic autorefraction, these threshold-shift analyses were also interpreted as a robustness check against potential boundary misclassification around the high-myopia cutoff. A stable performance profile under stricter SE definitions provides indirect evidence that the model was not solely driven by borderline cases near −6.00 D, although this analysis cannot replace validation against cycloplegic refraction.

**Table 11:** Sensitivity analysis of EGS-Net across alternative high-myopia thresholds.

SE threshold	Positive cases, *n*	AUC	Sensitivity	Specificity	F1-score
≤ −6.00 D	162	0.9994	0.9533	0.9972	0.9369
≤ −6.50 D	122	0.9992	0.9486	0.9975	0.9264
≤ −7.00 D	91	0.9991	0.9421	0.9978	0.9146

### 3.5 Clinical utility and generalizability

An examination of samples correctly identified by EGS-Net but missed by baseline models revealed that these individuals often had “moderate” baseline refraction but adverse historical trajectory signals (e.g., < −1.25 D/year). By prioritizing kinetic trajectory over static status, EGS-Net acts as a proactive screening tool for future high-myopia risk. The intentional increase in sensitivity provides a critical window for preemptive clinical intervention before irreversible vision loss occurs.

To evaluate the clinical generalizability of EGS-Net, we performed a stratified analysis across different age-based developmental stages (Table 12). Historical refractive trajectory patterns relevant to future high-myopia risk often differ between early childhood (6–9 years) and early adolescence (10–15 years).

**Table 12:** Performance consistency of EGS-Net across age subgroups.

Age group	Sample size	AUC	Recall	F1-score
Early childhood (6–9y)	1,420	0.9991	0.9621	0.9214
Pre-adolescence (10–12y)	2,150	0.9995	0.9512	0.9402
Early adolescence (13–15y)	1,403	0.9992	0.9455	0.9381

**Table 13:** Comparison of model calibration (brier score).

Model	Brier score	Expected calibration error (ECE)
XGBoost	0.0421	0.0315
Random forest	0.0485	0.0382
**EGS-Net (ours)**	**0.0212**	**0.0144**

Bold values indicate the best performance metrics within each evaluation category.

The results demonstrate that EGS-Net maintains high performance across all age groups. Notably, the highest Recall (0.9621) was achieved in the 6–9y group, which is the most critical window for early intervention. This suggests that the knowledge-augmented module effectively captures adverse historical trajectory patterns associated with elevated future high-myopia risk in younger children.

In clinical screening, the reliability of predicted risk probabilities is as important as binary classification accuracy. We compared the Brier Score (where lower values indicate better calibration) of EGS-Net against standalone models.

EGS-Net achieved the lowest Brier Score (0.0212), indicating that the risk probabilities provided by our framework are more closely aligned with true clinical prevalence. This calibration is essential for providing actionable risk assessments to clinicians and parents.

To assess the robustness of EGS-Net against temporal shifts in screening data, we implemented a time-aware validation analysis in which model development used earlier screening-derived trajectories, while evaluation was performed on later eligible follow-up records according to the same student-level temporal-boundary rule. EGS-Net retained an AUC of 0.9982 and a Recall of 0.9410, demonstrating that the learned trajectory patterns and heuristic rules are stable over time and not overfitted to specific screening cycles. This analysis was intended as a robustness check rather than a replacement for the primary student-level cross-validation protocol.

## 4 Discussion

Overall, this study proposed and validated **EGS-Net**, a knowledge-augmented machine learning framework designed to predict future high-myopia risk within school-based longitudinal screening programs. By synthesizing baseline refractive status with trajectory-derived features—most notably the annualized SE progression rate—our framework achieved a high and clinically favorable **Recall of 0.9533**. This outcome demonstrates that integrating clinical heuristics as a “safety net” effectively addresses the inherent sensitivity limitations of purely data-driven models while maintaining a substantially higher precision than Naive Bayes.

In terms of model performance, although standalone boosting algorithms like AdaBoost and XGBoost achieved high AUC and Precision, they exhibited a notable “sensitivity gap,” failing to identify a significant portion of students who later met the high-myopia endpoint (Recall ≈ 0.82). Although Naive Bayes yielded the numerically highest Recall, its very low Precision (0.4211) would generate an impractically large number of false-positive alerts. In a public health context, the clinical cost of a false negative (failing to flag a child who later meets the high-myopia endpoint) is high, but excessive false positives can also overburden referral pathways; therefore, the more meaningful advantage of EGS-Net lies in its stronger balance between sensitivity and precision.

The favorable clinical performance profile of **EGS-Net** is attributed to its dual-layer architecture. The **Stacking Layer** captures non-linear interactions between age, visual acuity, and refraction, while the **Clinical-Heuristic Override Module** explicitly targets the long-tail subgroup of students whose historical refractive trajectories indicate elevated future high-myopia risk. As shown in our ablation study, this expert-guided mechanism reclaimed nearly 9% of students previously missed by baseline risk-prediction models, proving that hybrid models can be more robust for high-stakes medical screening than standard “black-box” ensembles.

From a causal perspective, younger age, lower baseline spherical equivalent, and faster annual progression rate were independent risk factors for high myopia. Targeted interventions include: (1) closer monitoring for children under 10 years old, with shortened screening intervals for high-risk students; (2) timely clinical referral and individualized intervention for those with progression faster than −1.0 D/year; and (3) lifestyle guidance emphasizing increased outdoor time, reduced continuous near-work, and regular visual-rest breaks to slow myopia progression.

For real-world school-health deployment, transparency is a critical requirement. By employing SHAP (SHapley Additive exPlanations), we identified that **Baseline SE** and **Annualized Progression Rate** are the primary drivers of risk. Interestingly, the model captured a synergistic effect: younger age groups (< 10 years) combined with moderate progression rates triggered higher risk attributions than older students with similar progression. This aligns with the clinical principle that early onset combined with adverse historical trajectory increases future high-myopia risk, allowing EGS-Net to provide explainable alerts that clinicians and parents can trust.

In relation to prior work, existing myopia prediction literature has often relied on cross-sectional data or static baseline snapshots, although several recent studies have begun to incorporate machine learning and richer longitudinal or behavioral information (10–14, 16, 42). Our work extends this line of research by demonstrating that the **temporal trajectory** of refraction contains predictive signals that are not fully captured by baseline status alone. Rather than optimizing accuracy alone, our framework explicitly prioritizes **Recall together with clinical usability**, providing a more actionable tool for real-world school surveillance where repeated measures are available but often underutilized. This design is aligned with recent guidance for AI-based prediction model reporting and transparency (17, 19, 43–46).

Notably, recent student-population studies have shown that behavior-linked predictors and ensemble learners can provide meaningful early-warning value in non-clinical settings (47, 48). A recent large-scale study involving more than 40,000 children and adolescents further underscored the value of time-aware deep learning and longitudinal trajectory information for myopia prediction (49). In comparison with that black-box deep learning framework, EGS-Net may offer greater clinical interpretability and operational safety by incorporating explicit expert heuristic rules, while preserving balanced and stable performance for future high-myopia risk identification. Our longitudinal school-screening design is consistent with this direction, but further incorporation of structured behavioral covariates is still needed to improve individualized intervention recommendations.

Despite these strengths, this study has several limitations. First, the current results are based on a school-screening cohort from a single county in Yunnan Province, China. Although the dataset reflects a real-world public-health screening setting, differences in ethnicity, socioeconomic background, educational pressure, screening protocols, and health-care access may limit direct generalization to other regions. External validation using independent cohorts from different geographic and clinical settings is therefore required before broad deployment.

Second, given the real-world operational nature of large-scale school-based screenings, this study relied on non-cycloplegic autorefraction. This approach is feasible for routine campus screening but may introduce measurement bias or misclassification related to active accommodation, particularly in younger children. Because an independent cycloplegic gold-standard sub-sample was unavailable, non-cycloplegic refractive measurements could not be directly calibrated against the clinical gold standard. We therefore interpret EGS-Net as a school-screening risk-stratification tool rather than a diagnostic substitute for cycloplegic refraction. The high-myopia threshold sensitivity analysis (−6.00, −6.50, and −7.00 D) provides a statistical robustness check under shifting clinical boundaries and potential cutoff-related noise, but future studies should include cycloplegic sub-samples or external clinical cohorts to further verify predictive transportability.

Third, several important behavioral, familial, and environmental determinants of myopia were not available in the routine screening database, including outdoor activity, near-work duration, screen exposure, parental myopia, genetic susceptibility, and household or school-level environmental factors. The absence of these variables may restrict the model's ability to capture modifiable risk mechanisms and may partly explain residual prediction errors. Future implementations should integrate structured behavioral and familial questionnaires or school-environment indicators to support more individualized intervention recommendations.

Fourth, because this was a retrospective analysis of routine screening records, the dataset may contain operational sources of bias, including inconsistent follow-up intervals, student absenteeism, measurement variability, incomplete data upload, and missing values. Although we used within-student interpolation and cross-validation-nested MICE imputation to reduce information loss and avoid leakage, imputation cannot fully eliminate bias if the missingness mechanism deviates from the assumed missing-at-random pattern. Therefore, prospective data collection with standardized follow-up intervals and predefined quality-control procedures is needed to strengthen causal interpretation and operational reliability.

Finally, EGS-Net was intentionally designed to prioritize recall in order to reduce false negatives among students who later met the high-myopia endpoint. This design may increase false-positive alerts and referral burden compared with more conservative classifiers. Therefore, the decision threshold and expert-rule override parameters should be recalibrated according to local clinical capacity, referral pathways, and acceptable trade-offs between missed cases and unnecessary follow-up.

Future research will focus on incorporating environmental factors (e.g., near-work time and outdoor activity) and investigating probability calibration techniques, such as Platt scaling, to provide students with a “risk probability score” rather than a binary label.

In conclusion, the **EGS-Net** framework represents a shift from purely data-driven AI to **knowledge-augmented intelligence** in myopia screening. By bridging the gap between ensemble learning and clinical expertise, our approach provides a scalable, high-sensitivity, and interpretable solution for identifying children at risk of high myopia. This framework supports the transition toward personalized, proactive vision care in school-based public health settings.

## Data Availability

The raw data supporting the conclusions of this article will be made available by the authors, without undue reservation.
